# Beyond Platelet Count: Rethinking Platelet-Rich Plasma Efficacy Through Growth Factor Biology and Functional Quality

**DOI:** 10.3390/life16020188

**Published:** 2026-01-23

**Authors:** Fábio Ramos Costa, Joseph Purita, Rubens Martins, Luyddy Pires, Ansar Mahmood, Gabriel Silva Santos, André Kruel, João Protásio Netto, José Fábio Lana

**Affiliations:** 1 Department of Orthopedics, FC Sports Traumatology, Salvador 40296-210, BA, Brazil; 2 PUR-FORM, Boca Raton, FL 33432, USA; jpurita@aol.com; 3 Medical School, Tiradentes University Center, Maceió 57038-000, AL, Brazil; rubensdeandrade@hotmail.com; 4 Department of Orthopedics, Brazilian Institute of Regenerative Medicine (BIRM), Indaiatuba 13334-170, SP, Brazil; luyddypires@gmail.com (L.P.); kruel.andre@gmail.com (A.K.); josefabiolana@gmail.com (J.F.L.); 5 Regenerative Medicine, Orthoregen International Course, Indaiatuba 13334-170, SP, Brazil; 6 Trauma and Orthopaedics Department, University Hospitals Birmingham NHS Foundation Trust, Queen Elizabeth Hospital, Birmingham B15 2GW, UK; ansar.mahmood@uhb.nhs.uk; 7 Department of Orthopedics, Federal University of Tocantins (UFT), Palmas 77001-090, TO, Brazil; jprotasionetto@gmail.com; 8 Medical School, Max Planck University Center (UniMAX), Indaiatuba 13343-060, SP, Brazil; 9 Clinical Research, Anna Vitória Lana Institute (IAVL), Indaiatuba 13334-170, SP, Brazil; 10 Medical School, Jaguariúna University Center (UniFAJ), Jaguariúna 13911-094, SP, Brazil

**Keywords:** platelet-rich plasma, growth factors, platelet quality, regenerative medicine, standardization

## Abstract

The efficacy of platelet-rich plasma (PRP) has long been associated with platelet concentration, yet clinical outcomes remain highly variable and frequently inconsistent. This review challenges the assumption that platelet count alone defines PRP efficacy, proposing instead that functional platelet quality and growth-factor bioactivity are equally critical determinants of therapeutic outcomes. Platelets act as carriers of bioactive molecules stored within alpha granules, including growth factors such as platelet-derived growth factor (PDGF), transforming growth factor beta (TGF-β), vascular endothelial growth factor (VEGF), and insulin-like growth factor (IGF), which orchestrate the cellular and molecular events of tissue repair. Variations in donor biology, age, metabolic status, and oxidative stress profoundly influence platelet functionality and growth-factor release. Likewise, centrifugation parameters, temperature control, and activation methods dictate whether these mediators are preserved or prematurely exhausted. Collectively, these findings reveal that platelet number alone cannot predict regenerative potency. The future of PRP standardization requires the integration of platelet quality indices, growth-factor quantification, and patient optimization protocols into clinical practice. By shifting focus from platelet enumeration to bioactivity assessment, regenerative medicine can achieve more consistent, personalized, and scientifically accurate outcomes.

## 1. Introduction

Platelet-rich plasma (PRP) has evolved from a fringe autologous concentrate into one of the most widely adopted regenerative therapies across orthopedics, sports medicine, dermatology, and dentistry. By delivering endogenous bioactive molecules directly to sites of tissue injury, PRP has offered a biologically intuitive and minimally invasive alternative when compared with some conventional pharmacological and surgical interventions [1]. Yet, despite its widespread use and growing mechanistic evidence base, the field continues to be hampered by inconsistency in clinical outcomes and by the persistent assumption that total platelet number alone defines therapeutic potency. This limited view, which equates platelet number with regenerative efficacy, has become deeply ingrained in both clinical practice and industry marketing, fostering a culture of “more is better” that overlooks the underlying biological reality of PRP function.

This misconception arises from an early but incomplete understanding of platelet biology. Platelets are not therapeutic in their physical abundance; they are transient carriers of molecular information stored within their alpha granules and other vesicular compartments [2]. Platelets are anucleate cells containing specialized organelles, including alpha and dense granules that store bioactive mediators, as well as mitochondria that support platelet metabolism and contribute to activation-dependent functional responses [3]. Their clinical value depends on the integrity and release dynamics of these bioactive reservoirs, which include a complex array of growth factors, cytokines, and signaling mediators that orchestrate angiogenesis, cellular proliferation, matrix remodeling, and immune modulation [2,4]. As a result, a preparation with fewer but functionally competent platelets can exert more potent regenerative activity than one containing a higher number of exhausted or senescent cells [5].

Recent investigations into platelet senescence have further reinforced this principle. Evidence indicates that chronological age, systemic inflammation, and oxidative stress degrade platelet quality long before any measurable decline in count occurs [5]. Senescent platelets show altered ultrastructure, premature degranulation, and dysregulated cytokine profiles that may compromise, or even counteract, the intended regenerative effect [5]. These findings challenge long-standing assumptions about dose dependency in orthobiologic therapies and expose a fundamental disconnect between quantitative protocols and functional biology. The previously published concept of platelet aging highlights that platelet concentration, while measurable and reproducible, fails to capture the molecular competence that determines real-world efficacy.

Parallel advances in growth factor quantification have provided direct biochemical support for this paradigm shift. Studies measuring growth factors found in PRP samples demonstrate that inter-individual variability in PRP bioactivity is driven more by donor biology and processing conditions than by raw platelet counts [6,7]. The same number of platelets can yield dramatically different concentrations of growth factors depending on the patient’s age, metabolic profile, centrifugation parameters, and activation method [5,6,7]. This evidence collectively suggests that regenerative potential resides in biochemical quality rather than numerical abundance.

**Figure 1:**
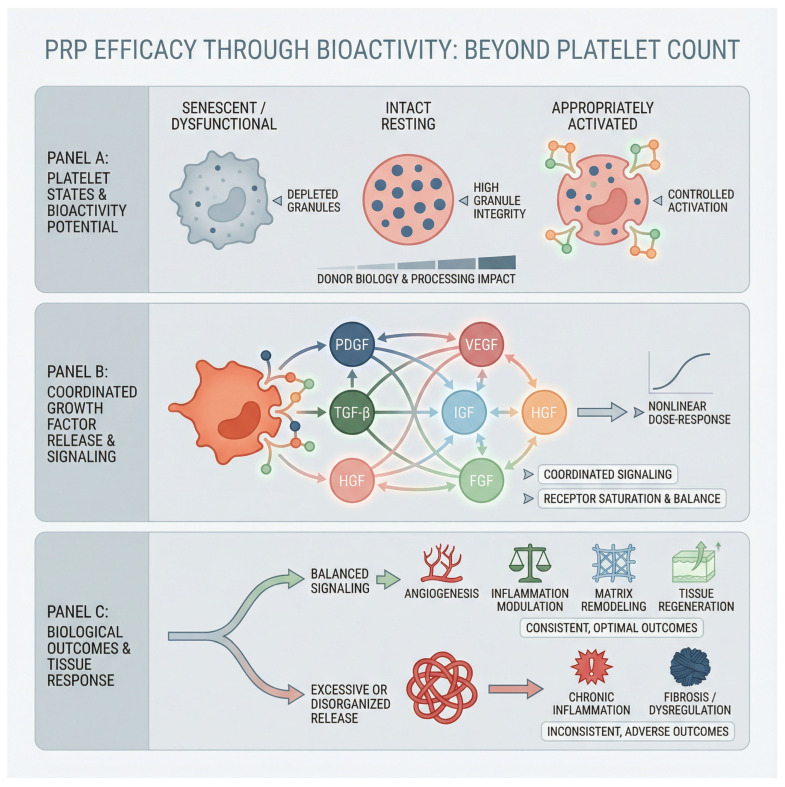
Systems-level model of platelet-rich plasma efficacy based on functional bioactivity rather than platelet count.

Taken together, these observations demand a reassessment of how PRP efficacy is conceptualized, standardized, and evaluated. The focus must transition from a count-based model toward a bioactivity-based framework grounded in measurable growth factor content, platelet functional quality, and target tissue responsiveness. Such a shift carries immediate implications for patient selection, processing protocols, and clinical trial design, while also laying the foundation for a new generation of standardized, biologically meaningful PRP formulations. In essence, the future of PRP therapy depends not on how many platelets are delivered, but on how effectively they communicate the regenerative message encoded within their molecular cargo. The integrated relationship between platelet state, coordinated growth factor signaling, and downstream biological outcomes is summarized in Figure 1.

## 2. The Historical Fixation on Platelet Numbers

The early development of PRP therapy coincided with the rapid expansion of point-of-care centrifugation systems, each competing to market its own proprietary definition of “richness.” As platelet enumeration was the simplest measurable variable, platelet count quickly became the default surrogate for potency. Multiplicative terms such as “three-fold” or “five-fold” concentration were adopted as marketing shorthand for biological superiority, creating an illusion of standardization in a field still largely empirical. Clinical enthusiasm, bolstered by anecdotal success and reinforced by the apparent logic of “more platelets, more healing,” cemented this numerical paradigm as dogma [5].

Yet this focus on absolute platelet numbers overlooked the most fundamental principle of regenerative biology: efficacy is determined by the bioactive molecules released, not by the cellular containers themselves. The platelet is not the drug; it is the delivery system [5]. Studies reporting associations between platelet dose and outcomes often remain vulnerable to confounding biological and procedural factors. As the field matured, the limitations of this quantitative fixation became increasingly apparent. Two PRP preparations with identical platelet concentrations could exhibit entirely different profiles of growth factors, cytokines, and extracellular vesicles, resulting in divergent biological responses [5].

It is important to emphasize that platelet count should not be dismissed as clinically irrelevant. Meta-analyses have demonstrated that platelet concentration contributes to outcomes within specific therapeutic windows, and preparations falling below minimum thresholds may lack sufficient cellular substrate for adequate growth-factor delivery [8,9]. The critique presented here targets the reductionist assumption that platelet number alone predicts efficacy, and not the inclusion of platelet count as one component of quality assessment. Rather than abandoning enumeration, we advocate for its integration with functional parameters, recognizing platelet count as a necessary but not sufficient condition for regenerative potency.

The obsession with achieving ever-higher platelet yields has also influenced procedural practice in ways that contradict basic hematologic and physiologic reasoning. Large-volume blood draws, often exceeding 100 to 200 mL, are justified on the assumption that higher platelet input guarantees greater output. However, this approach neglects the finite distribution of platelets within circulating blood and disregards the qualitative degradation that can occur during prolonged processing [5]. Centrifugation protocols employing high relative centrifugal force (RCF), prolonged spin duration and repeated mechanical stress induce partial activation, premature degranulation, and shear-related membrane injury, thereby reducing the availability of intact, growth-factor-rich platelets for therapeutic use [10,11,12]. Ironically, the quest for quantity may directly compromise the very quality that governs regenerative performance.

Moreover, the narrative of platelet count supremacy has propagated inconsistencies across both research and clinical translation. Studies have reported highly variable results despite nominally similar platelet concentrations, exposing the weakness of count-based standardization [8,13]. Commercial platelet-rich plasma devices frequently advertise quantitative enrichment without parallel disclosure of platelet viability or growth-factor yield. In parallel, existing regulatory frameworks do not always stipulate performance metrics related to platelet function, resulting in broad uncertainty regarding the true therapeutic quality of the end product. As a consequence, clinicians are left comparing numerical metrics divorced from biological meaning, and patients receive treatments whose efficacy depends more on unseen biochemical dynamics than on any measurable platelet ratio.

Several classification systems have been proposed to describe and categorize platelet-rich plasma preparations, primarily based on parameters such as platelet concentration, leukocyte content, activation status, and fibrin architecture. These frameworks were developed to facilitate reporting consistency and comparability across studies and clinical applications. However, while useful for descriptive purposes, most existing classification approaches focus on structural or compositional features and do not capture functional platelet quality, growth factor bioactivity, or donor-related biological variability. Consequently, PRP preparations that fall under similar classification labels may exhibit substantial differences in biological potency. The main PRP classification systems and their defining characteristics are summarized in Table 1.

In light of these realities, the field stands at an inflection point. Continuing to define PRP by platelet concentration alone perpetuates methodological noise and clinical unpredictability. True standardization requires shifting attention from enumeration to function. Only by abandoning the historical fixation on numbers can PRP science advance toward genuine biological precision.

## 3. Growth Factors as the True Therapeutic Agents

The therapeutic essence of PRP lies not in the presence of platelets but in the molecular payload they carry. Each platelet is a transient vehicle containing pre-synthesized signaling proteins that govern every stage of tissue repair [5]. Within their alpha granules resides a remarkably diverse library of growth factors, cytokines, chemokines, and matrix-modifying enzymes that act in coordinated sequence once released [4]. The biological potency of any PRP formulation therefore depends on the integrity, concentration, and release kinetics of these mediators rather than on the numerical abundance of their carriers.

Alpha granules hold the majority of the platelet’s regenerative content. Among the most relevant molecules are platelet-derived growth factor (PDGF), transforming growth factor beta (TGF-β), vascular endothelial growth factor (VEGF), epidermal growth factor (EGF), insulin-like growth factor (IGF), fibroblast growth factor (FGF), and hepatocyte growth factor (HGF) [4]. Together they drive angiogenesis, stimulate fibroblast and stem-cell proliferation, regulate extracellular matrix synthesis, and modulate inflammation [4]. Dense granules contribute additional mediators that influence local hemostasis and immune activity, while lysosomal vesicles release enzymes that remodel the extracellular environment [14,15,16]. This molecular complexity explains why PRP cannot be reduced to a single quantitative variable. The principal growth factors stored within platelet alpha granules and their corresponding biological actions are summarized in Table 2.

Quantitative assays indicate that platelet count alone fails to predict growth-factor output [6]. Measurements using ELISA and multiplex cytokine arrays reveal wide inter-individual variability in the amount of PDGF, TGF-β, and VEGF released per million platelets. As discussed earlier, donor and processing factors modify this yield independently of platelet number [6,7,34,35,36]. Therefore, even the same patient could produce distinct bioactive profiles from two PRP samples processed with slightly different protocols. These findings expose a fundamental limitation of platelet enumeration as a quality metric and highlight the need for direct biochemical assessment of PRP content.

The biological functions of these growth factors further illustrate why therapeutic success depends on their balance rather than on platelet concentration. For instance, PDGF recruits fibroblasts and smooth-muscle cells to the injury site and initiates the proliferation phase of repair [37]. Beyond its well-established pleiotropic roles summarized in Table 2, TGF-β signaling in PRP primarily influences the balance between matrix remodeling and fibrotic responses in a context-dependent manner [18]. In PRP formulations, VEGF contributes chiefly to the orchestration of angiogenic signaling cascades, with its functional impact shaped by concentration, release kinetics, and interaction with other growth factors rather than by isolated abundance [38]. IGF and EGF accelerate cellular proliferation and migration, while FGF and HGF contribute to matrix formation and epithelial repair [4,39]. Each factor acts within a narrow concentration window, and their combined ratios determine whether the healing response is regenerative or fibrotic. Delivering an excessive amount of platelets without ensuring appropriate growth-factor balance elevates the risk of producing an incoherent biochemical signal that the tissue cannot interpret effectively. This aligns with evidence indicating that the relationship between platelet concentration and regenerative outcomes is inherently nonlinear. As highlighted in a recent study [9], PRP efficacy reflects the complex regulatory behavior of the cytokine network, with optimal biological activity occurring within a defined concentration window (approximately 600–900 × 10^9^/L). Concentrations above this range do not necessarily enhance clinical outcomes; instead, they likely trigger a saturation effect at the level of growth-factor–receptor interactions. Once this threshold is exceeded, increasing platelet numbers may contribute to signaling noise, diminishing the coordination of downstream pathways and ultimately impairing the regenerative response. In addition to platelet-derived growth factors, PRP contains other cellular components that can modulate its biological activity, both positively and negatively, as summarized in Table 3.

While this review emphasizes platelet-derived growth factors as primary mediators of regeneration, the contribution of leukocytes to PRP bioactivity warrants explicit consideration. L-PRP formulations contain varying concentrations of neutrophils, monocytes, and lymphocytes, each contributing distinct signaling molecules and enzymatic activities. Some authors suggest that leukocytes enhance antimicrobial defense and provide additional cytokines that may amplify early inflammatory signaling necessary for tissue repair initiation [36]. In contrast, other reports indicate that elevated neutrophil content increases matrix metalloproteinase (MMP) activity and catabolic cytokine release, potentially counteracting the anabolic effects of platelet-derived growth factors [40,41].

Clinical evidence remains divided. In tendinopathy and muscle injuries, some studies suggest improved outcomes with P-PRP formulations, attributing this to reduced inflammatory burden at already inflamed tissue sites [13,36]. In contrast, intra-articular applications for osteoarthritis show more heterogeneous results, with certain trials reporting comparable efficacy between L-PRP and P-PRP [9]. The optimal leukocyte content likely depends on tissue type, pathology chronicity, and the baseline inflammatory status of the target environment.

From a bioactivity perspective, leukocyte inclusion adds another layer of compositional variability that current platelet-centric frameworks fail to capture. Future standardization efforts should therefore report not only leukocyte concentration but also differential counts and activation states, enabling more precise correlation with clinical outcomes. The ongoing L-PRP versus P-PRP debate reinforces the central thesis of this review: that compositional metrics alone, whether platelet- or leukocyte-based, cannot substitute for functional bioactivity assessment.

Beyond their canonical roles in wound healing, platelet-derived molecules participate in immunomodulation and neuroregeneration. Cytokines such as interleukin-4 and interleukin-10 help shift macrophages toward a pro-resolutive “M2” phenotype [32,33], while neurotrophins such as BDNF and NGF add a neuromodulatory dimension to PRP bioactivity [29,30,31]. These actions are governed by concentration thresholds and receptor sensitivities rather than by platelet dose alone.

Understanding PRP through the lens of growth-factor biology transforms it from a crude concentrate into a molecularly precise therapy. It compels clinicians and researchers to evaluate the qualitative attributes of the platelet secretome and to optimize preparation methods that preserve this fragile biochemical cargo. The next frontier in standardization will not rely on counting platelets but on quantifying the molecules that give them purpose.

## 4. Variables Affecting Growth-Factor Yield

The regenerative output of PRP is shaped as much by the donor’s biology as by the preparation technique. Even when identical protocols are applied, growth-factor composition varies widely among individuals because platelet function reflects systemic physiological status rather than isolated hematologic metrics [5].

**Age**

Age remains the most consistent determinant of platelet bioactivity. With advancing age, megakaryocytic efficiency declines and the proportion of senescent platelets in circulation increases [5,42,43]. As introduced earlier, platelet senescence is associated with a progressive decline in PRP efficacy, driven by structural alterations and reduced granule integrity that compromise growth-factor release [5,42,43]. Concurrently, age-related cellular changes in target tissues may further limit responsiveness to these mediators. A preparation derived from an elderly or metabolically compromised donor may therefore contain the same platelet count as that of a young individual but deliver a fraction of the effective biological signal [5].

**Metabolic Status**

Metabolic status exerts a similar, often underappreciated influence. Chronic hyperglycemia, dyslipidemia, and insulin resistance generate oxidative stress that accelerates platelet turnover and exhausts antioxidant defenses [5,44]. The resulting platelets are hyperreactive yet inefficient, releasing growth factors prematurely and in disorganized patterns [43,45]. This biochemical disarray may partially explain the inconsistent outcomes observed in patients with metabolic syndrome or poorly controlled diabetes [46]. In these contexts, systemic correction of oxidative imbalance may yield greater benefit than adjustments in centrifugation speed or platelet dose.

**Inflammation**

Since PRP mirrors the patient’s physiological milieu, both inflammation and hormonal status can also shape its composition. Interleukin(IL)-6 and Tumor Necrosis Factor alpha (TNF-α) drive pro-inflammatory platelet phenotypes [40,41], whereas sex-steroid deficiency blunts growth-factor synthesis, as evidenced by post-menopausal women, for instance, who display lower PDGF-BB levels linked to reduced estradiol [47]. These variations highlight the need for patient profiling rather than reliance on platelet counts alone.

**Processing and Handling**

Processing time and handling conditions further affect donor biology because delays between blood collection and activation accelerate metabolic decay within platelets [48,49]. These effects may be amplified in older or inflamed patients whose platelets already show signs of senescence and oxidative stress [5]. Temperature fluctuations compound this impact by promoting spontaneous activation and early degranulation [48,49]. Even under controlled laboratory settings, these biological susceptibilities can shift the biochemical fingerprint of PRP within relatively short timeframes [48,49]. Anticoagulant selection represents an additional processing variable that may influence platelet activation status during PRP preparation. Citrate-based anticoagulants are commonly used to transiently inhibit coagulation during handling [50]; however, the absence of standardized recommendations and the heterogeneity of preparation systems [51] limit the generalizability of anticoagulant-specific effects, placing this factor outside the primary scope of the present review.

Recognizing these patient-specific variables reframes PRP therapy as a biologically dynamic intervention rather than a standardized product. The success of treatment depends not only on technical precision but on optimizing the biological starting material. Strategies such as antioxidant supplementation, lifestyle modulation, and pre-procedural metabolic stabilization should therefore be viewed as integral components of regenerative protocols. Quality control must begin with the donor before it can be achieved in the tube [5,52,53].

## 5. Technique Matters: How Processing and Activation Define Biology

Once the biological potential of platelets is acknowledged, the next determinant of PRP quality lies in how those platelets are handled. Every stage of preparation, from venipuncture to activation, has measurable consequences for structural integrity, growth-factor preservation, and final bioactivity. The technical process is not merely logistical; it is a form of biological modulation.

The most influential variable is centrifugation. The entire purpose of spinning blood is to concentrate viable platelets while maintaining the delicate architecture of their membranes and granules. In practice, however, high RCF protocols designed to maximize platelet yield can produce the opposite effect; platelets are subjected to high mechanical stress that ruptures membranes and triggers premature degranulation [54]. Even when platelet counts appear elevated after processing, a significant fraction of these cells may have already released their alpha-granule contents, yielding a concentrate rich in empty shells rather than functional mediators [5,54,55]. Lower RCF, longer duration protocols tend to preserve morphology and sustain growth-factor reservoirs, resulting in a biologically competent product even when the numerical yield is modest [56,57].

Temperature adds another layer of control. Platelets are metabolically active fragments, and deviations from physiologic conditions alter their enzymatic and secretory behavior [58,59,60]. Processing at room temperature favors spontaneous activation and metabolic drift, whereas controlled short-term cold storage (2–6 °C) can preserve platelet structure and hemostatic potential [49]. Prolonged refrigeration or repeated temperature fluctuations, however, disrupt calcium homeostasis and cytoskeletal organization, ultimately impairing platelet responsiveness upon rewarming [61].

The interval between blood draw and activation is equally critical. Prolonged delays permit progressive decline in mitochondrial potential and increase the proportion of pre-activated platelets [61,62]. These time-dependent changes explain why identical systems can produce divergent clinical results depending on workflow discipline. A standardized window, ideally within 1 h, should be treated as essential quality control rather than procedural convenience [63].

The activation method defines both the kinetics and the biological relevance of growth-factor release. Exogenous activators such as thrombin and calcium chloride trigger platelet degranulation but differ greatly in tempo. Thrombin provokes rapid and almost complete exocytosis of alpha granules, generating an intense yet short-lived burst of mediators [64]. Calcium chloride induces a slower and more sustained release that reflects the physiological rhythm of tissue repair [64]. In contrast, endogenous activation occurs when platelets come into contact with native collagen and other extracellular matrix components at the site of application [64]. This interaction produces a gradual and prolonged release of growth factors that enhances matrix integration and extends biological activity in vivo [64]. Selecting an activation pathway should therefore depend on the biological objective: rapid hemostasis, prolonged angiogenesis, or sequential remodeling rather than relying on habit or convenience.

Other procedural details often overlooked can alter the molecular profile of the final product. Anticoagulant choice affects platelet pH and calcium homeostasis, thereby modulating baseline activation status, viability, and potential for growth-factor release [35,65]. Repetitive pipetting or vortex mixing can introduce turbulence that triggers partial activation [66]. Even the geometry and material of the collection tube can alter shear distribution during centrifugation, influencing fibrin architecture and the recovery of growth factors in the final product, which in turn further complicates efforts toward standardization [67]. These technical nuances may appear trivial, yet together they decide whether the resulting PRP acts as a coherent regenerative signal or a chaotic cocktail of degraded proteins.

Understanding technique as a form of biological stewardship reframes laboratory precision as a central therapeutic variable. The clinician’s goal should be to preserve the latent regenerative potential of the platelet, not to maximize counts or volumes. A preparation produced under gentle, physiologic conditions and activated in a controlled manner will almost invariably deliver a more consistent and biologically effective result than a numerically richer but mechanically exhausted counterpart. Technique, in this sense, is biology in disguise.

## 6. From Numbers to Function—A Framework for Future Standardization

The evolution of PRP therapy has reached a critical inflection point. For decades, progress was defined by technical refinements that promised higher platelet yields, shorter spin times, and greater fold-increase ratios. Yet these innovations, though technologically impressive, have not translated into consistent biological outcomes. The reason lies in the equivocal assumption that platelet number always equates to potency. The emerging evidence dismantles that notion and replaces it with a more realistic view: PRP efficacy depends on the molecular competence of the platelets and the biological environment into which they are introduced. Recognizing this principle requires a redefinition of what constitutes quality in PRP formulations. A conceptual comparison between platelet count–based and bioactivity-based interpretations of PRP efficacy is summarized in Table 4.

Emerging clinical evidence supports the translation of bioactivity-based concepts into patient outcomes. A recent systematic review and meta-analysis demonstrated that PRP outcomes in knee osteoarthritis are associated with the total amount of deliverable platelets rather than concentration alone [8]. Conversely, in lateral epicondylitis, platelet concentration did not significantly affect pain outcomes, suggesting that additional biological variables govern therapeutic response in tendinopathy [13]. Comparative trials evaluating leukocyte-poor versus leukocyte-rich formulations suggest that the inflammatory profile of the preparation influences both short-term symptom relief and long-term tissue remodeling [36]. Studies measuring growth factor levels have revealed substantial inter-individual variability in PDGF, TGF-β, and VEGF concentrations that correlates more strongly with donor age, sex, and baseline platelet characteristics than with final platelet counts [6,7]. Critically, investigations that standardized preparation protocols while measuring growth factor output have achieved more consistent outcomes than those relying solely on platelet enumeration [9,36]. While definitive dose-response relationships for individual growth factors remain to be established, the cumulative evidence reinforces the principle that functional characterization improves predictive accuracy and clinical reproducibility.

Platelet concentration is a convenient metric because it is easy to measure, but it is not biologically meaningful in isolation. A truly functional classification must integrate both the intrinsic quality of the platelet population and the biochemical potency of its secretome. Cellular integrity and molecular yield should form the basis of any future standardization framework. Quantifying platelet viability, mitochondrial function, or membrane integrity alongside direct assays of growth factor concentrations would provide a more complete picture of therapeutic capacity [5]. Such a system would capture the essential principle that the regenerative potential of PRP lies not in numerical abundance but in preserved bioactivity [5]. A practical standardization framework should therefore incorporate platelet concentration as a baseline parameter while layering functional metrics, such as viability indices and growth-factor yields, to provide a more complete biological profile. This dual approach preserves the logistical simplicity of enumeration while enhancing predictive accuracy through bioactivity assessment.

Several initiatives have been proposed to improve reporting quality and reproducibility in PRP research, primarily through standardized documentation of compositional and procedural parameters such as platelet concentration, leukocyte content, activation status, and preparation methods. These efforts have contributed to improved transparency and comparability across studies. However, most reporting frameworks remain largely descriptive and do not account for platelet functional quality, growth factor bioactivity, or nonlinear signaling behavior, which may partially explain persistent variability in biological and clinical outcomes. Integrating functional and bioactivity-oriented parameters alongside existing reporting criteria may therefore represent an important next step in PRP standardization.

Moving in this direction also demands that patient-specific factors be treated as part of product quality control. Patient-specific physiology influences platelet biology before the first drop of blood is drawn [5,52,53]. Conditioning protocols that enhance redox balance and cellular metabolism could therefore improve PRP composition as effectively as any device modification. Integrating pre-procedural optimization frameworks such as nutritional correction, metabolic control, and lifestyle adjustment into clinical workflows may become a critical feature of next-generation orthobiologic practice. Preparation should begin with the patient, not the tube [5,52,53].

Advances in analytical technology will accelerate this shift. Quantitative immunoassays and multiplex proteomic platforms are now capable of measuring key growth factors and other bioactive proteins with high precision in plasma-derived products [68]. Miniaturized or point-of-care devices may soon allow rapid profiling of PRP bioactivity directly in the clinic, transforming what is now an empirical intervention into a measurable and reproducible therapy. Such tools would enable real-time adjustment of processing parameters to achieve targeted molecular signatures, marking the transition from generic concentrate to personalized regenerative formulation.

Future classification systems should evolve beyond platelet enumeration toward bioactivity indices that describe functional performance. Metrics such as “growth factor per platelet ratio,” “alpha-granule release efficiency,” or “platelet quality index” could form the next generation of quality markers. These values would integrate biological, technical, and patient variables into a unified expression of regenerative potential. As these concepts mature, regulatory frameworks must adapt to acknowledge functional biomarkers as legitimate measures of product identity and potency.

To operationalize this framework in routine practice and future trials, a pragmatic starting point is to report a minimal quality control panel that captures composition, handling, and bioactivity. At the point of care, this can include baseline whole blood values, final platelet concentration together with leukocyte and erythrocyte contamination, the centrifugation protocol, the activation strategy, and the interval between collection and application. In research settings or higher fidelity clinical programs, adding a limited set of functional readouts such as platelet viability or activation markers and a small growth factor panel, for example, PDGF, TGF-β, VEGF, and IGF, can provide an interpretable biochemical signature that can be correlated with outcomes. Reporting these measures alongside clinical endpoints allows studies to move beyond numerical descriptors and to test whether signaling balance, rather than platelet abundance, predicts response across indications.

**Figure 2:**
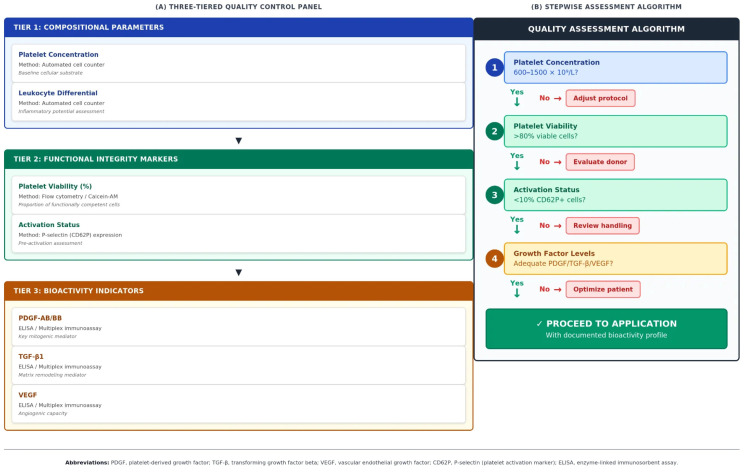
Proposed framework for biologically meaningful PRP quality assessment.

Based on the evidence reviewed, we propose a minimal quality control panel for biologically meaningful PRP characterization, summarized in Table 5 and schematically illustrated in Figure 2. This panel integrates three tiers: (1) compositional parameters (platelet concentration, leukocyte content), (2) functional integrity markers (platelet viability, membrane integrity), and (3) bioactivity indicators (key growth factor concentrations). Implementation may be adapted according to clinical setting and available resources, with point-of-care viability testing representing the most accessible entry point for functional assessment.

The three-tiered evaluation (left panel) integrates compositional parameters (Tier 1), functional integrity markers (Tier 2), and bioactivity indicators (Tier 3). The decision algorithm (right panel) provides a stepwise quality assessment protocol with corrective actions for suboptimal results at each stage. Abbreviations: PDGF, platelet-derived growth factor; TGF-β, transforming growth factor beta; VEGF, vascular endothelial growth factor; CD62P, P-selectin; GF, growth factor.

Rather than proposing rigid clinical protocols, the framework presented here is intended to guide biologically meaningful interpretation and reporting of PRP formulations. Emphasis on platelet functional quality, growth-factor bioactivity, and patient biological context provides a flexible foundation for future standardization efforts, allowing adaptation across indications and study designs. As methodological tools for functional assessment continue to evolve, this conceptual shift may facilitate more consistent translation of PRP therapies into clinical practice.

The integration of bioactivity-based metrics into regulatory frameworks presents both opportunities and challenges. In most jurisdictions, autologous PRP is classified as a minimally manipulated homologous-use product, exempt from the extensive premarket approval required for advanced therapy medicinal products (ATMPs). However, this classification assumes that the therapeutic action is attributable to the native properties of the cells rather than to defined molecular potency. As PRP evolves toward bioactivity-guided formulations, regulatory frameworks may increasingly emphasize demonstration of consistent potency, analogous to the release criteria applied to cell therapy products.

Current Good Manufacturing Practice (GMP) principles already emphasize process validation, reproducibility, and in-process controls, all of which align conceptually with functional quality metrics. Point-of-care PRP preparation typically occurs outside formal GMP environments, but the principles of documented standard operating procedures, equipment qualification, and batch-to-batch consistency remain applicable. The challenge lies in defining practical, validated potency assays suitable for clinical settings without imposing prohibitive analytical burdens.

International guidance documents, including those from the U.S. Food and Drug Administration (21 CFR Part 1271) and the European Medicines Agency (Regulation 1394/2007), increasingly emphasize functional characterization for cell-based therapies. Adopting even a minimal bioactivity panel, such as viability assessment and one or two key growth factor concentrations, would represent a feasible first step toward harmonized quality standards. Industry stakeholders, regulatory scientists, and clinical researchers should collaborate to establish consensus thresholds and reference standards that balance scientific rigor with practical implementation.

Ultimately, this transformation extends beyond methodology. It represents a philosophical shift in regenerative medicine, from extraction to precision, from counting to understanding. PRP should no longer be viewed as a blood derivative but as a living biological communication system whose success depends on message fidelity. Clinicians and researchers who embrace this perspective will unlock a new level of predictability and therapeutic control. The future of orthobiologic science will belong not to those who extract more blood, but to those who extract more meaning from it.

## 7. Conclusions

The traditional reliance on platelet count as the defining parameter of PRP efficacy has reached its limit. Decades of empirical refinement have failed to resolve the inconsistency in clinical outcomes because the central biological determinant has been misunderstood. Platelets are not therapeutic by their number but by their molecular content and functional quality. Growth factors, cytokines, and extracellular mediators stored within intact alpha granules constitute the real agents of regeneration.

This review integrates current evidence demonstrating that platelet function declines with age, metabolic dysfunction, and oxidative stress, while processing techniques further modify their bioactive potential. The clinical implication is clear: a higher platelet yield does not necessarily lead to consistently better outcomes, and excessive processing may even compromise regenerative efficacy. The field must transition toward a biologically grounded model in which growth factor composition, platelet integrity, and patient-specific variables are treated as measurable determinants of quality.

Future standardization should move beyond enumeration to incorporate indices of platelet bioactivity and functionality. Integrating these parameters into research and clinical protocols will produce more predictable results and elevate PRP from an empirical practice to a reproducible biologic therapy. The path forward in regenerative medicine lies not in increasing platelet numbers but in refining the molecular precision of what they deliver.

## Figures and Tables

**Table 1:** Major Classification Systems for Platelet-Rich Plasma.

Classification Axis	Representative Systems	Defining Criteria	Strengths	Key Limitations
Platelet concentration	P-PRP vs. L-PRP; concentration fold increase	Absolute or relative platelet count	Simple, quantitative	Ignores platelet functionality and bioactivity
Leukocyte content	Leukocyte-rich vs. leukocyte-poor PRP	Presence or absence of leukocytes	Accounts for inflammatory contribution	Does not distinguish leukocyte subtypes or activation state
Activation status	Activated vs. non-activated PRP	Use of calcium, thrombin, or endogenous activation	Influences release kinetics	Poor standardization of activation protocols
Fibrin architecture	PRP vs. PRF variants	Liquid vs. fibrin matrix	Relevant for scaffolding applications	Limited relevance to signaling quality
Composite systems	PAW, DEPA, MARSPILL	Multivariable scoring	Improved reporting structure	Still largely descriptive, not bioactivity-driven

Abbreviations: PRP, platelet-rich plasma; P-PRP, pure platelet-rich plasma; L-PRP, leukocyte-rich platelet-rich plasma; PRF, platelet-rich fibrin; PAW, platelet concentration–activation–white blood cell content; DEPA, dose–efficiency–purity–activation; MARSPILL, method–activation–red blood cells–spin–platelet concentration–image guidance–leukocytes–light activation.

**Table 2:** Principal growth factors contained in platelet-rich plasma and their primary biological actions.

Growth Factor	Major Target Cells	Primary Biological Actions	Key Contribution to Regeneration	Key References
PDGF (Platelet-Derived Growth Factor)	Fibroblasts, smooth muscle cells, mesenchymal progenitors	Stimulates proliferation, chemotaxis, and extracellular matrix production	Initiates tissue repair and granulation formation	[17]
TGF-β (Transforming Growth Factor Beta)	Fibroblasts, chondrocytes, immune cells	Regulates collagen synthesis, controls inflammation, and promotes matrix remodeling	Coordinates transition from inflammation to remodeling	[18]
VEGF (Vascular Endothelial Growth Factor)	Endothelial cells, pericytes	Induces angiogenesis and increases vascular permeability	Enhances oxygenation and nutrient supply to regenerating tissue	[19]
EGF (Epidermal Growth Factor)	Epithelial cells, keratinocytes, fibroblasts	Promotes re-epithelialization and cell migration	Accelerates wound closure and tissue coverage	[20]
IGF-1 (Insulin-Like Growth Factor 1)	Myoblasts, fibroblasts, osteoblasts	Stimulates protein synthesis, cell proliferation, and differentiation	Supports muscle, bone, and connective-tissue regeneration	[21,22,23]
FGF (Fibroblast Growth Factor)	Fibroblasts, chondrocytes, endothelial cells	Drives fibroblast proliferation and angiogenesis	Facilitates connective-tissue repair and cartilage regeneration	[24,25]
HGF (Hepatocyte Growth Factor)	Epithelial and mesenchymal cells	Modulates cell motility and morphogenesis; exerts anti-fibrotic effects	Encourages organized tissue regeneration and reduces scarring	[26,27,28]
BDNF (Brain-Derived Neurotrophic Factor)	Neurons, Schwann cells	Promotes neuronal survival and axonal growth	Contributes to peripheral nerve healing and sensory recovery	[29]
NGF (Nerve Growth Factor)	Neurons, glial cells	Enhances neurite outgrowth and remyelination	Improves neuroregenerative capacity in peripheral tissues	[30,31]
EGF-like peptides and Cytokines (IL-4, IL-10)	Immune and stromal cells	Shift macrophage polarization toward anti-inflammatory phenotypes	Supports immune resolution and tissue remodeling	[32,33]

**Table 3:** Cellular Constituents of Platelet-Rich Plasma and Their Biological Roles.

Component	Key Subtypes/Features	Biological Contribution	Potential Beneficial Effects	Potential Detrimental Effects
Platelets	Functional vs. senescent platelets; alpha and dense granules	Storage and regulated release of growth factors, cytokines, and chemokines	Angiogenesis, matrix remodeling, cell proliferation, tissue repair	Reduced efficacy with senescence, premature degranulation, oxidative stress
Leukocytes	Neutrophils, monocytes, lymphocytes	Immune modulation, antimicrobial activity, cytokine signaling	Early inflammatory signaling, debris clearance, immune regulation	Excess inflammation, protease release, catabolic signaling
Erythrocytes	Residual contamination	Oxygen transport (minimal relevance in PRP)	Limited or none	Oxidative stress, hemolysis-derived toxicity, inflammatory amplification
Extracellular vesicles	Platelet- and leukocyte-derived microvesicles and exosomes	Paracrine signaling, RNA and protein delivery	Fine-tuning of regenerative signaling, intercellular communication	Poorly characterized variability, potential pro-inflammatory effects

**Table 4:** PRP Efficacy Interpreted by Molecular Composition Rather Than Platelet Count.

Evaluation Parameter	Platelet Count-Based Interpretation	Bioactivity-Based Interpretation
Primary metric	Absolute platelet number	Growth factor yield, ratios, and release kinetics
Biological assumption	More platelets produce stronger effects	Balanced signaling determines response
Dose–response behavior	Linear	Nonlinear with saturation thresholds
Predictive value	Low for clinical outcomes	Higher when functional parameters are considered
Clinical consistency	Highly variable	Improved when bioactivity is optimized
Standardization potential	Limited	Supports biologically meaningful standardization

**Table 5:** Proposed Minimal Quality Control Panel for PRP Characterization.

Tier	Parameter	Measurement Method	Rationale
1 (Composition)	Platelet concentration	Automated cell counter	Baseline cellular substrate
1 (Composition)	Leukocyte differential	Automated cell counter	Inflammatory potential assessment
2 (Functional)	Platelet viability (%)	Flow cytometry/calcein-AM	Proportion of functionally competent cells
2 (Functional)	Activation status	P-selectin (CD62P) expression	Pre-activation assessment
3 (Bioactivity)	PDGF-AB/BB concentration	ELISA/multiplex immunoassay	Key mitogenic mediator
3 (Bioactivity)	TGF-β1 concentration	ELISA/multiplex immunoassay	Matrix remodeling mediator
3 (Bioactivity)	VEGF concentration	ELISA/multiplex immunoassay	Angiogenic capacity

## Data Availability

No new data were created or analyzed in this study.
